# A History of the Work "Principles and Practice of Dentistry"

**Published:** 1885-03

**Authors:** 


ARTICLE III.

### A HISTORY OF "THE PRINCIPLES AND PRACTICE OF DENTISTRY."

*Including Anatomy, Physiology, Pathology, Therapeutics, Dental Surgery and Mechanism.*—By Chapin A. Harris, M. D., D. D. S. Eleventh edition. Revised and edited by Ferdinand J. S. Gorgas, A. M., M. D., D. D. S., author of "Dental Medicine," Professor of the Principles of Dental Science, Dental Surgery and Dental Mechanism in the University of Maryland. With two full-page plates and 744 other illustrations. 994 pages. Octavo. Cloth, $6.50; Leather, $7.50. Philadelphia: P. Blakiston, Son & Co., 1885.

[From the April No. 1885, of the *Independent Practitioner*.]

"The publication of the eleventh edition of the oldest of our standard text-books demands something more than a passing notice. It marks an era in our professional literature. We use the term advisedly and after due consideration, for the last decade has witnessed such an advance in the knowledge upon which an intelligent practice if founded, that its first crystallization into our permanent literature may well be considered as a distinctive epoch.

To Chapin A. Harris is universally conceded the position of Father of Modern American Dentistry. He did more to give it its distinctive character, to mark out its future course, to mould and fashion it into what it is to-day than any other man. That American Dentistry differs from the dentistry of any other country, is mainly due to his writings and teachings. When medical schools were closed against the teachings of dental science, Harris, with his coadjutors organized an independent college, instituted a new and peculiar degree, and launched dentistry in its own bark. Under the peculiar circumstances of the time there was no other course to take, if we were not to degenerate into mere handicraft.

The first edition of The Principles and Practice of Dentistry was published by Dr. Harris in 1839, the same year in which he founded THE AMERICAN JOURNAL OF DENTAL SCIENCE, the first dental journal published, and one year before he established the first dental college in the world

It was a thin, octavo volume, with but a few illustrations but it very fairly represented the then infant science of dentistry. Although conducting a large private practice, he was constantly engaged in literary work. The publishing of books and journals was not then reduced to a kind of science, as it is to-day, and for many years Dr. Harris was his own publisher. He conducted his private practice, wrote and published books, and edited, and a part of the time published, a monthly journal. How would he have regarded some of the men of to-day, who, with a practice no larger than his own, and nothing else to occupy their time complain that they have not the necessary leisure for even reading dental journals, to say nothing of a comprehensive study of the literature of their profesion.

The second edition of one thousand copies of this work was, by agreement with Dr. Harris, published in

Philadelphia, in 1844, by Lindsay & Blakiston, the predecessors of the present publishers. This edition contained many new illustrations, and was considerably increased in size and comprehensiveness. The third edition, of the same number of copies, was published in 1847. The fourth edition, of one thousand copies, was published in 1850. The fifth, in 1852; the sixth, in 1855; the seventh, in 1858; the eighth, in 1863; the ninth in 1866. Each of the last five of these editions consisted of fifteen hundred copies, and each was more or less revised and enlarged to meet the rapid advances made by the growing science.

Dr. Harris having died in 1860, and the status of the profession demanding that the work be thoroughly revised and enlarged for the ninth edition, Dr. Philip S. Austin, now deceased, was selected to do the work. He was assisted by Prof. F. J. S. Gorgas in the departments of pathology and surgery, and by Dr. Thomas S. Latimer in anatomy and physiology. Each of these men had been associated with Dr. Harris as a teacher in the Baltimore College of Dental Surgery, and under their joint care the tenth edition, of which five thousand copies have been sold, was issued in 1871. Very much of the work was entirely rewritten, and considerable additions were made to the text.

This edition sufficed for the wants of the profession for some years, but as the copies upon the shelves of the publishers gradually disappeared, it became evident that yet another addition must be prepared. Since the publication of the tenth edition, wonderful advances had been made in some of the fields of dental science. The histology of 1870 had become a thing of the past. Recent discoveries, by careful observers, had shown that some of the teachings of the tenth edition were erroneous. In pathology the advances had been most marked, and the book had, even in the few years that had elapsed, become almost antiquated. The teachings of 1870 would not answer for 1880, much less for 1885. In Mechanics and prosthesis the change in practice had not been so marked, but even here careful revision was neccessary. The work of preparing the new edition was entrusted to Prof. F. J. S. Gorgas, one of the editors of the preceding edition, as the man best qualified in all respects for the labor, and the consequence is a book that may almost be called a cyclopedia of dental practice. So great is the contrast with the tenth edition, that it may be considered practically a new book. Much of the matter in the old edition has been struck out as obsolete, or erroneous, yet so large have been the additions that the new work exceedes the size of the old by two hundred pages. Four hundred new illustrations have been added, and many of these are the most valuable that the work contains. To-day, Harris' Principles and Practice is is the most complete text-book known to the profession.

In addition to the sale of the former editions in this country, the book has received a large and steady demand from the profession in England, and it has been translated into French. Including the present edition, eighteen thousand five hundred copies have been printed, a most unprecedented sale for a work of this character. The amount paid in royalties to Dr. Harris and his heirs has amounted to a very large sum, and the copyright money is still paid them.

In writing this sketch of his principal work, it may not be amiss to include a few facts concerning the honored author, for every dentist should know something of the life of one so prominent in his profession.

Chapin A. Harris was born at Pompey, Onondaga Co., N. Y., in 1806. He graduated in medicine in 1830, and for a few years was a general practitioner, when he turned his attention to dentistry. Besides his continual contributions to the AMERICAN JOURNAL OF DENTAL SCIENCE, he wrote a "Dictionary of Medicine and Dental Surgery," of which five editions have been published, and he translated from the French a number of works of importance. He died in September, 1860, at the age of fifty-four, from a disease contracted through overwork.

When we set out to write this notice, it was with the intention of presenting a digest of the contents of the eleventh edition of Dr. Harris' great work, and to this end copious notes were made during its examination, and it was carefully compared with the ninth and tenth editions. But limited space forbids the pursuance of this plan, nor is it necessary. It is sufficient to say that the book is brought up fully abreast the advance of thought, and to-day we may take an honest pride in the fact that dentistry possesses such a text-book. Instead of the teachings of Goodsir, and Robertson, and Beil, we have the later discoveries and observations of Kolliker, and Waldeyer, and Miller. In every department it is made complete, and in harmony with the most modern accepted theories. It is an essential in the library of every dental student, and is quite as necessary to the possessors of the earlier editions as to those who have never seen them.

It is needless to say that the publishers have done their full duty, and that the letter press, illustrations and binding, are perfect of their kind.

[From the *Dental Cosmos*, March No. 1885.]

The first edition of this book was published in 1841; the last (tenth) edition was issued under the editorship of the late Dr. P. H. Austen.

Nearly fourteen years having elapsed since that revision, the rapid advances made during that period have necessitated another, which, as stated in the preface, was undertaken by Prof. Gorgas at the request of the publishers and of the author's family, involving, as he claims, more than a year's labor. Many changes have been made in the arrangement of topics; new chapters have been added; the number of illustrations has been greatly increased, and much that had become obsolete in theory and practice has been omitted.

Of the omissions, it is only necessary to say that good judgment has been exercised in expurgation.

Of the many valuable additions, the most considerable are under the heads of irregularity of the teeth, preparation of natural roots for attachment of artificial crowns, and vulcano-plastic work; but many other notable and valuable additions have been made through-out the volume.

The book is divided into four parts, treating respectively of anatomy and physiology, pathology and therapeutics, dental surgery, and dental mechanics. The literature of the profession has evidently been diligently searched, and we can scarcely recall a plausible theory which has been suggested, or an improved appliance or method which has been devised, within the last decade, that has not received more or less notice by the editor. There has evidently been an earnest and conscientious effort to incorporate in this volume all that belongs to a text-book of dentistry. It is sufficiently elementary for the student, while at the same time a reliable guide to the practitioner.

This new edition of Harris is so far ahead of all previous editions that it should be in the possession of every dental student, and there are few practitioners who can afford not to number it among their possessions.

It would not be difficult to criticise some of the teaching. No volume of this extent was ever written which was not open to criticism, and none such will ever be written that, in all respects, will find indorsement by any one individual. But, in the main, the instruction conveyed is reliable, the methods taught are such as are approved by some at least of those who are considered as authorities; and, until the times comes when all authorities shall agree upon all points, universal indorsement is not to be expected. We think, however, that all unprejudiced readers will admit that the eleventh edition of Harris is a volume of which the dental profession may not feel ashamed.

DR. B. H. CATCHING, editor of *The Southern Dental Journal*, April No. 1885, writes as follows concerning the new edition: "Dr. Gorgas has added greatly to the value of the work." "It now comes to us fully up to the present status of the profession." "The work as now revised stands far in the lead of any other work on dentistry," Dr. Gorgas spent a year's hard work in accomplishing what to any mind was a difficult undertaking. "That he has done his work well is evident, and an appreciative profession will accord him all praise." "As the work stands it is the text book for the student and a guide to the experienced." "Every library should have a copy of this new edition."

The following appears in the *Archives of Dentistry*, May No. 1885, from Dr. C. W. Spalding, editor.

"To say that this edition is greatly superior to either of its predecessors, is to only do simple justice to the editor. The laborious task of revising this large volume has in the main been well done and the result is highly creditable to the head and hand that has done the work." "The publishers have done their part and all concerned in the production of this eleventh edition of Harris deserve the sincere thanks of both students and practitioners."

Dr. George Watt, editor of the *Ohio State Journal of Dental Science*, writes as follows in the May No. 1885. "This splendid book is undoubtedly *the* work on modern dental practice and reflects great credit on its editor—we are tempted to say author, for it is virtually a new work by Prof. Gorgas." "We have carefully examined the thousand pages and find little or nothing in them to criticise." "Every reader of the *Journal* should own a copy for reference, if not for study."

